# Factors affecting the spread of multiple information in social networks

**DOI:** 10.1371/journal.pone.0225751

**Published:** 2019-12-12

**Authors:** Zhiqiang Zhu, Yinghao Zhang

**Affiliations:** College of Science, Huazhong Agricultural University, Wuhan, Hubei, China; Beijing University of Posts and Telecommunications, CHINA

## Abstract

Information spreading in social networks is affected by many factors. Based on a novel information spreading model with five spreading mechanisms, we analyzed and compared the influence of various factors on information spreading. Through a large number of simulation experiments, we found that: (1) *K*-*shell* layers have the greatest impact on information spreading; (2) distance between the two information sources, correlation coefficient between two types of information and social reinforcement also affect the information spreading. The analysis results of these factors will be helpful for us to predict the trend of information spreading and find effective strategies to control information spreading.

## Introduction

With the rapid development of Internet technology, online social networks have gained increasing popularity, and the application scope is becoming broader. In the past decade, information spreading in social networks has attracted great research interests [1–12].

Unlike the spread of disease, the spread of information will leave certain memories of the information on the individuals once they received it. Whenever an individual receives one new information, social reinforcement will to some extent affect the acceptance of the information by the individual: when he receives the information for many times, the possibility of accepting this information will increase. Under the influence of memory and social reinforcement, information will be more easily accepted by people. When people receive one information, their attention to this information will decline over time, which will decrease the effects of memory and social reinforcement, and reduce the coverage of information spreading. In a word, information spreading may be influenced by memory, social reinforcement and attentional decline. In addition, in the first contact with the information, the degree of acceptance of the information may varies from individual to individual. So information spreading may be also largely influenced by “human heterogeneity”.

Dodds and Watts [13] introduced “memory effect” into the process of information spreading, and discovered that it can affect the spread of information. Wu et al. [14] used a dynamic model to describe the relationship between the growth of news and the mechanism of “group attentional decline” in a website. Centola [15] found that “social reinforcement” can influence information spreading in an empirical research. Lü et al. [16] put forward a model that evaluates the essential differences between the spread of information and the spread of the epidemic, and the model contained three mechanisms of “memory effect”, “social reinforcement” and “non-redundant contact”. Nematzadeh et al. [17] used a threshold model as the dynamic model of information spreading, and the research showed that community structure has a significant impact on information spreading. Kitsak et al. [18] reported a model of single information spreading, and found that the source of information was located at the *K*-*shell* layer with the highest *K*_*S*_ value, which can contribute to a wider coverage of information spreading. Zhu et al. [19] first proposed the concept of “human heterogeneity” and a novel information spreading model that included four spreading mechanisms: “memory effect”, “social reinforcement”, “non-redundant contact” and “human heterogeneity”; besides, the influence of “human heterogeneity”, “social reinforcement” and network structure on the model of single information spreading was also analyzed.

Most current studies of information spreading focused on the spreading characteristics of single information. However, in real social networks, it is almost impossible that only one type of information is transmitted in a certain period of time. Instead, various types of information are present in the network at the same time, and in the process of spreading, there may be certain interactions among different types of information, which will certainly affect the whole spreading behavior. As a matter of fact, the simultaneous spreading of various types of information is a very complicated process. At present, the researches on multiple information spreading were mainly based on a variety of disease propagation models [20]. However, these models can not well reflect the “non-redundant contact” of information and ignore the spreading characteristics such as “memory effect”, “social reinforcement” and “attentional decline”.

In this paper, we proposed a novel information spreading model, which can simulate the multiple information spreading in the network. This model included the interaction between two types of information and five different spreading mechanisms: (1) memory effects, (2) social reinforcement, (3) non-redundant contact, (4) human heterogeneity and (5) attentional decline. Based on this model, we assessed the impacts of the spreading mechanisms, interaction between the two types of information, distance between the two information sources, community structure and *K*-*shell* layers on information spreading. The major aim of the paper is to analyze what factors affect the spread of information, and clarify which are the primary, secondary and unimportant factors.

## Materials and methods

The main research methods of this paper include: (1) build a new model of information spreading; (2) based on this spreading model, through simulation experiments to analyze the influence of various influencing factors on information spreading in social networks. In order to understand the model more clearly, the next two parts (“Basic Definitions” and “Process of Information Spreading”) explain the concepts and contents of the model. Then in “Preparations for Simulation Experiments”, the background networks of the simulation experiment and how to analyze the influence of various factors are described.

### Basic definitions

Suppose that two types of information *A* and *B* are propagated in network *G*. In the network, each node represents an individual, and each edge represents the social relationship between two individuals. Some of the related definitions in our model are as follows:

1. information attribute [*I*_*A*_, *I*_*B*_]: *I*_*A*_, *I*_*B*_ ∈ [0, 1] is a constant reflecting the degree of difficulty in accepting information *A* or *B* by individuals. A lower information attribute suggests that the information is more easily accepted.
2. individual attribute $[I_{A}^{\prime }\left(v\right),I_{B}^{\prime }\left(v\right)]:I_{A}^{\prime }\left(v\right)$, $I_{B}^{\prime }\left(v\right)\in [0,1]$ is a constant that reflects the degree to which individual *v* is willing to accept information, when he first receives information *A* or *B*. The larger the individual attribute is, the easier the information will be accepted.
3. individual state: At each time step, each individual is in one of the four states:
  - “*Unknown*”: the individual has not yet come into contact with the information.
  - “*Known*”: the individual has received the information, but is not willing to propagate it, possibly because he is suspicious of the authenticity of the information.
  - “*Accepted*”: the individual accepts the information and then propagates it to all his neighbors.
  - “*Exhausted*”: after propagating the information to his neighbors, the individual will lose his interests in it and never propagate this information again.
4. The coverage of information spreading: It is the proportion of individuals who accept information to all individuals.
5. The correlation coefficient between two kinds of information *β*: *β* ∈ [−1, 1].
  - 0 < *β* ≤ 1, which indicates that when an individual comes into contact with two types of information, one type of information can promote the acceptance of the other information by the individual. The greater the value of *β* is, the easier it is for individuals to accept information.
  −1 ≤ *β* < 0, which indicates that when an individual comes into contact with two types of information, one type of information may hinder the acceptance of the other type by the individual. A lower *β* value will more significantly hinder the individual’s acceptance of the information.
  - *β* = 0, which indicates that the two types of information do not affect each other.

The spreading mechanisms in our model are described as follows.

1. Memory effects [*M*_*i*_(*v*, *t*), *i* = *A*, *B*]: The individual *v* has come into contact with the information *i* for *M*_*i*_(*v*, *t*) times until the *t* time step. In the process of information spreading, as time goes on, once the individual *v* contacts with the information *i* again, the value of *M*_*i*_(*v*, *t*) is increased by 1.
2. Social reinforcement (*c*): The constant *c* (*c* > 0) reflects the social reinforcement effect. The larger the value of *c* is, the easier the information is accepted.
3. Non-redundant contact: In the spreading process, each link in the network is used at most once.
4. Human heterogeneity: The difference in individual attribute reflects the intensity of human heterogeneity. The greater the fluctuations of individual attribute is, the stronger human heterogeneity is.
5. Attentional decline: As time goes on, the novelty of the information declines, so the individual’attention to information also declines. The attentional decline factor [9] was presented as
  $$\begin{array}{c} d_{\Delta t}\;=\;e^{-0.4{\left(\Delta t\right)}^{0.4}}\;\;\in \;\;\left(0,\;1\right], \end{array}$$ (1)
  where Δ*t* indicates the total time step from the first time that the information is received to the current time point.

### Process of information spreading

**Fig 1:**
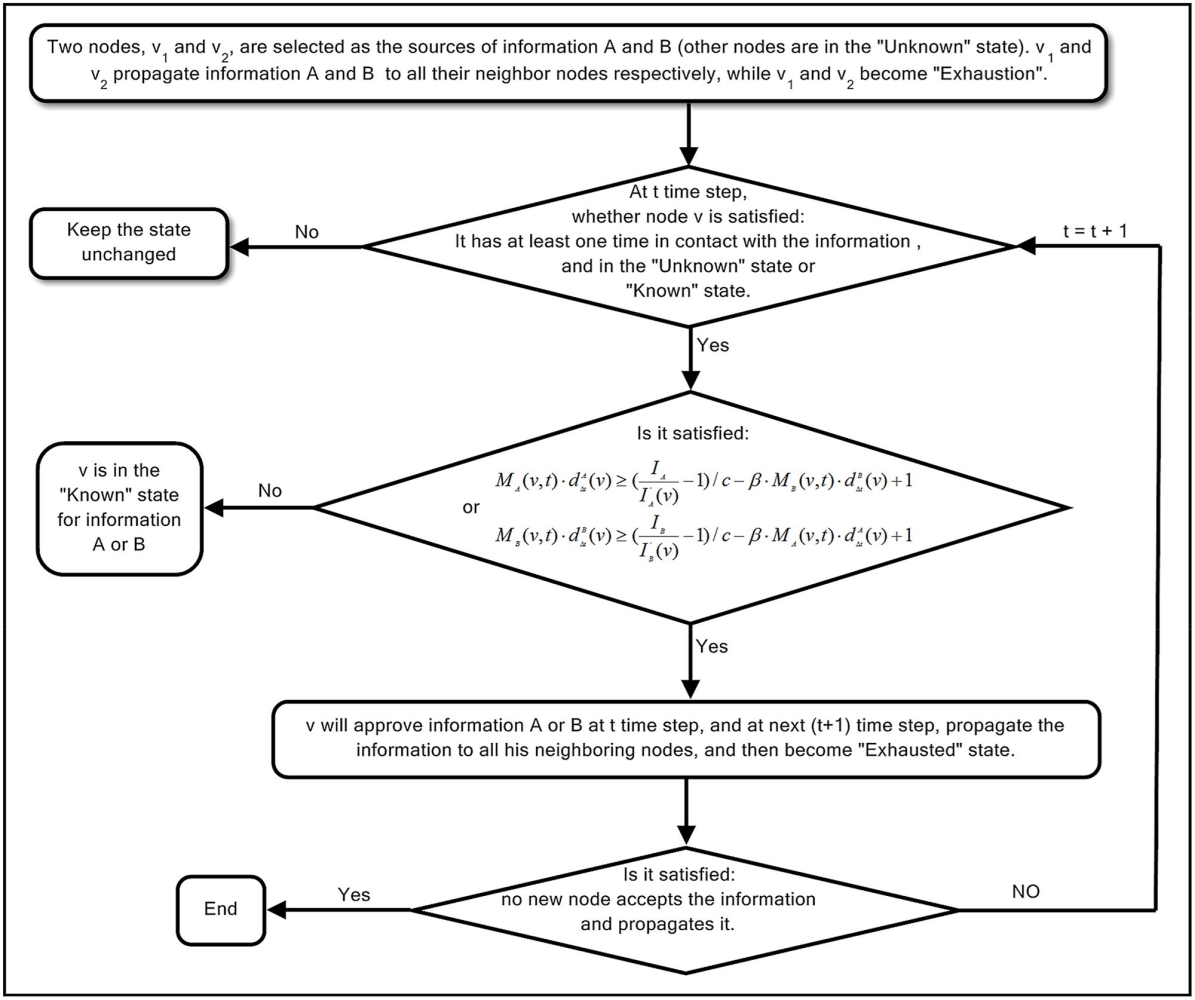
Spreading process of two types of information *A* and *B* in the network.

The spreading process of two types of information *A* and *B* in the network is shown in Fig 1.

The main steps in the process of information spreading are shown below:

Step 1 At the beginning, two nodes are randomly chosen as the information sources and the others are in the “*Unknown*” state. These sources will spread the information to all their neighbors and then turn to an “*Exhausted*” state.
Step 2 Once node *v* (in either an “*Unknown*” or a “*Known*” state for one of the two types of information) receives the information at least once at the *t* (*t* ≥ 1) time step, he will make judgments about its authenticity by the following principles.
  Suppose that node *v* has received information *A* for *M*_*A*_(*v*, *t*) times and information *B* for *M*_*B*_(*v*, *t*) times until *t* time step, the attentional decline factors of information *A* and information *B* are $d_{\Delta t}^{A}\left(v\right)$ and $d_{\Delta t}^{B}\left(v\right)$, respectively.
  (a) If $I_{A}\;\leq \;I_{A}^{\prime }\left(v\right)\left[c\left(M_{A}\left(v,t\right)\cdot d_{\Delta t}^{A}\left(v\right)\;+\;\beta \cdot M_{B}\left(v,t\right)\cdot d_{\Delta t}^{B}\left(v\right)-1\right)+1\right]$, *v* will accept information *A* at the *t* time step and spread it to all his neighbors in the next time step and then become an “*Exhausted*” state; otherwise, *v* will do nothing no matter how many times he comes into contact with information *A*.
  (b) If $I_{B}\;\leq \;I_{B}^{\prime }\left(v\right)\left[c\left(M_{B}\left(v,t\right)\cdot d_{\Delta t}^{B}\left(v\right)\;+\;\beta \cdot M_{A}\left(v,t\right)\cdot d_{\Delta t}^{A}\left(v\right)-1\right)+1\right]$, the conclusion of whether an individual accepts information *B* is similar to that in (a).
  However, if node *v* does not receive information *A* and *B* at the *t* time step, he will do nothing in the next time step.
Step 3 If no new individual accepts and spreads the information, the spreading process will come to an end; otherwise, the process will be continued as Step 2.

**Fig 2:**
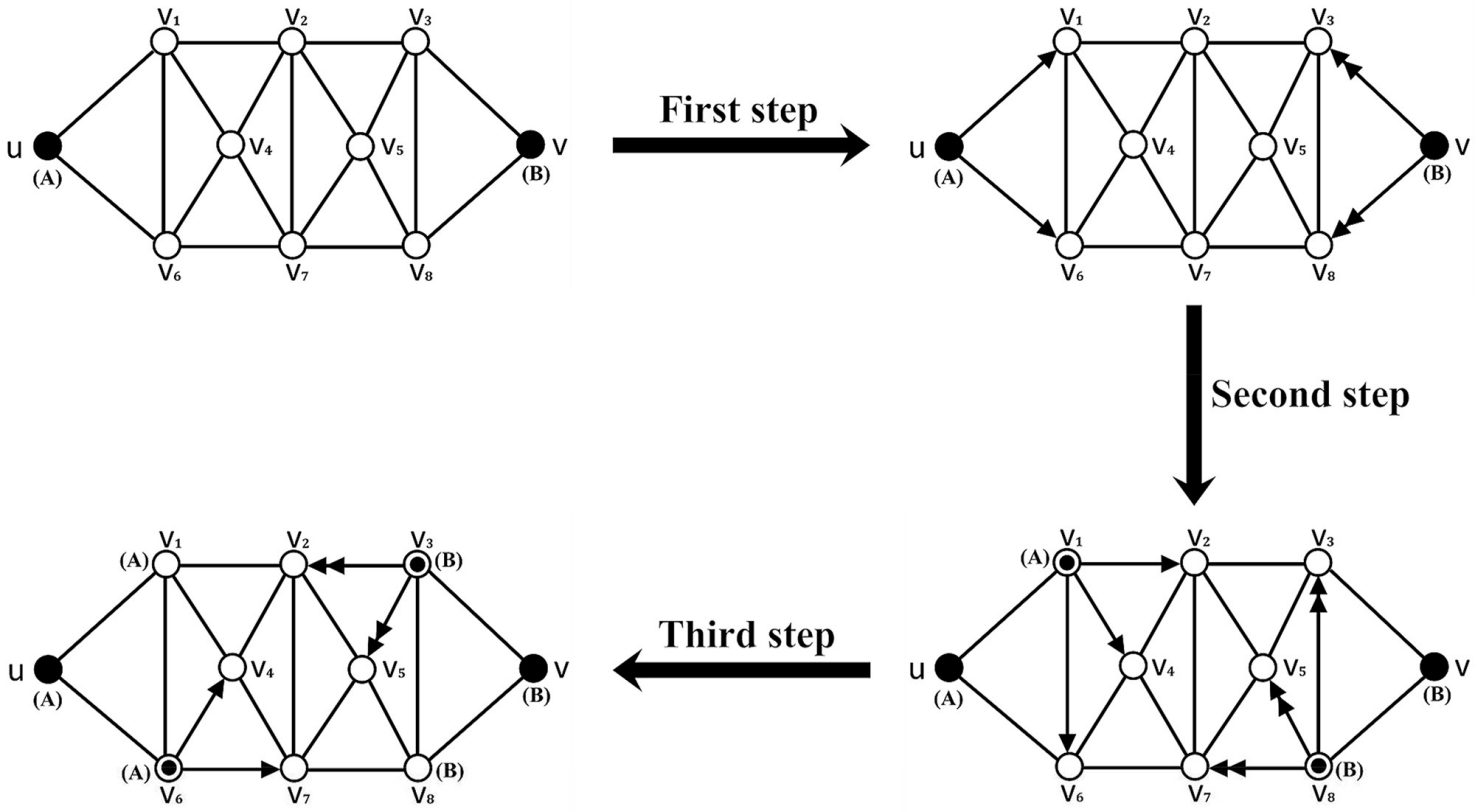
An example of the spreading process of two kinds of information *A* and *B* in network. Arrows indicate that information is transmitted from one node to another, and single arrow and double arrows represent the propagation of *A* and *B*, respectively.

In order to understand the above process of information spreading, we give an example (see Fig 2). Fig 2 is an example of the spreading process of two kinds of information *A* and *B* in network.

Firstly, *u* and *v* are the nodes where information *A* and *B* begin to spread, respectively. Let *I*_*A*_ = *I*_*B*_ = 0.6, $I_{A}^{\prime }\left(v_{1}\right)=I_{B}^{\prime }\left(v_{1}\right)=0.8$, $I_{A}^{\prime }\left(v_{2}\right)=I_{B}^{\prime }\left(v_{2}\right)=0.2$, $I_{A}^{\prime }\left(v_{3}\right)=I_{B}^{\prime }\left(v_{3}\right)=0.5$, $I_{A}^{\prime }\left(v_{4}\right)=I_{B}^{\prime }\left(v_{4}\right)=0.4$, $I_{A}^{\prime }\left(v_{5}\right)=I_{B}^{\prime }\left(v_{5}\right)=0.4$, $I_{A}^{\prime }\left(v_{6}\right)=I_{B}^{\prime }\left(v_{6}\right)=0.5$, $I_{A}^{\prime }\left(v_{7}\right)=I_{B}^{\prime }\left(v_{7}\right)=0.2$, $I_{A}^{\prime }\left(v_{8}\right)=I_{B}^{\prime }\left(v_{8}\right)=0.8$, and *c* = 1.0, *β* = 1.0.

Then information spreading includes three steps:

**First step**: $I_{A}^{\prime }\left(v_{1}\right)=0.8>0.6=I_{A}$, $I_{A}^{\prime }\left(v_{6}\right)=0.5<0.6=I_{A}$, $I_{B}^{\prime }\left(v_{3}\right)=0.5<0.6=I_{B}$, $I_{B}^{\prime }\left(v_{8}\right)=0.8>0.6=I_{B}$, then *v*_1_ accepts *A*, *v*_8_ accepts *B*.
**Second step**: $I_{A}^{\prime }\left(v_{2}\right)=0.2<0.6=I_{A}$, $I_{A}^{\prime }\left(v_{4}\right)=0.4<0.6=I_{A}$, $I_{A}^{\prime }\left(v_{6}\right)\left[\left(2e^{-0.4}-1\right)+1\right]=0.5\times 1.34=0.67>0.6=I_{A}$, $I_{B}^{\prime }\left(v_{3}\right)\left[\left(2e^{-0.4}-1\right)+1\right]=0.5\times 1.34=0.67>0.6=I_{B}$, $I_{B}^{\prime }\left(v_{5}\right)=0.4<0.6=I_{B}$, $I_{B}^{\prime }\left(v_{7}\right)=0.2<0.6=I_{B}$, then *v*_6_ accepts *A*, *v*_3_ accepts *B*.
**Third step**: $I_{A}^{\prime }\left(v_{4}\right)\left[\left(2e^{-0.4}-1\right)+1\right]=0.4\times 1.34=0.54<0.6=I_{A}$, $I_{A}^{\prime }\left(v_{7}\right)\left[\left(1+1-1\right)+1\right]=0.2\times 2=0.4<0.6=I_{A}$, $I_{B}^{\prime }\left(v_{2}\right)\left[\left(1+1-1\right)+1\right]=0.2\times 2=0.4<0.6=I_{B}$, $I_{B}^{\prime }\left(v_{5}\right)\left[\left(2e^{-0.4}-1\right)+1\right]=0.4\times 1.34=0.54<0.6=I_{B}$.

Finally, nodes *v*_1_ and *v*_6_ accept *A*, nodes *v*_3_ and *v*_8_ accept *B*.

### Preparations for simulation experiments

Based on the above spreading model, we then analyzed the influence of various factors in social network through a large number of simulation experiments. In this paper, we mainly carried out simulation experiments based on the following three kinds of networks:

1. a real social network (*G*_1_, the e-mail network of University Rovira i Virgili (URV) [21]): This network comprises more than 1000 nodes that represent faculty, researchers, technicians, managers, administrators, and graduate students, which considers the e-mails exchanged between university addresses during the first three months of 2002.
2. two networks (*G*_2_ and *G*_3_) generated by social network evolution model: In this paper, we chose the network evolution model in [22], which contains two adjustable parameters *p*_1_ and *p*_2_. Through the different values of these two parameters, we can get a network whose degree distribution obeys a certain distribution, where *G*_2_ is a network whose degree distribution obeys power law distribution (*p*_1_ = 0.1, *p*_2_ = 0.2), and *G*_3_ is a network that obeys stretched exponential distribution (*p*_1_ = 0.2, *p*_2_ = 0.3).
3. a real social network (*G*_4_, the network contains friendship data of Facebook users [23]): This network comprises 63,731 nodes and 817,035 edges. A node represents a user and an edge represents a friendship between two users. This dataset is obviously not complete and contains a very small subset of the total Facebook friendship graph.

**Fig 3:**
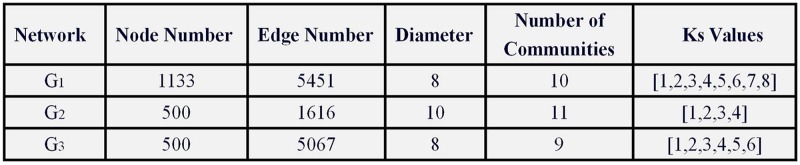
Some of the topological characteristics of three background networks.

Some of the topological characteristics of these four networks were listed in Fig 3. Here we used fast unfolding algorithm [24] and the *K*-*Shell* decomposition method [25] to obtain the community structure and the *K*-*Shell* layers of the network, respectively.

Next, we analyzed the simulation experiments of information spreading in *G*_1_, while the simulation experiments on network *G*_2_, *G*_3_ and *G*_4_ were in Supporting information (see S1, S2, and S3 Files).

## Results

Next, we chose the e-mail network of University Rovira i Virgili (URV) [21] as the background network of information spreading, and used the above spreading model to simulate and analyze the effects of social reinforcement, correlation coefficient between the two types of information, distance between the two information sources in the network, community structure and *K*-*shell* layers on information spreading. In the following simulation experiments, for “human heterogeneity”, we only considered the individual attributes that obey uniform distribution, and all results in this paper were obtained by averaging over 1,000 independent realizations.

**Fig 4:**
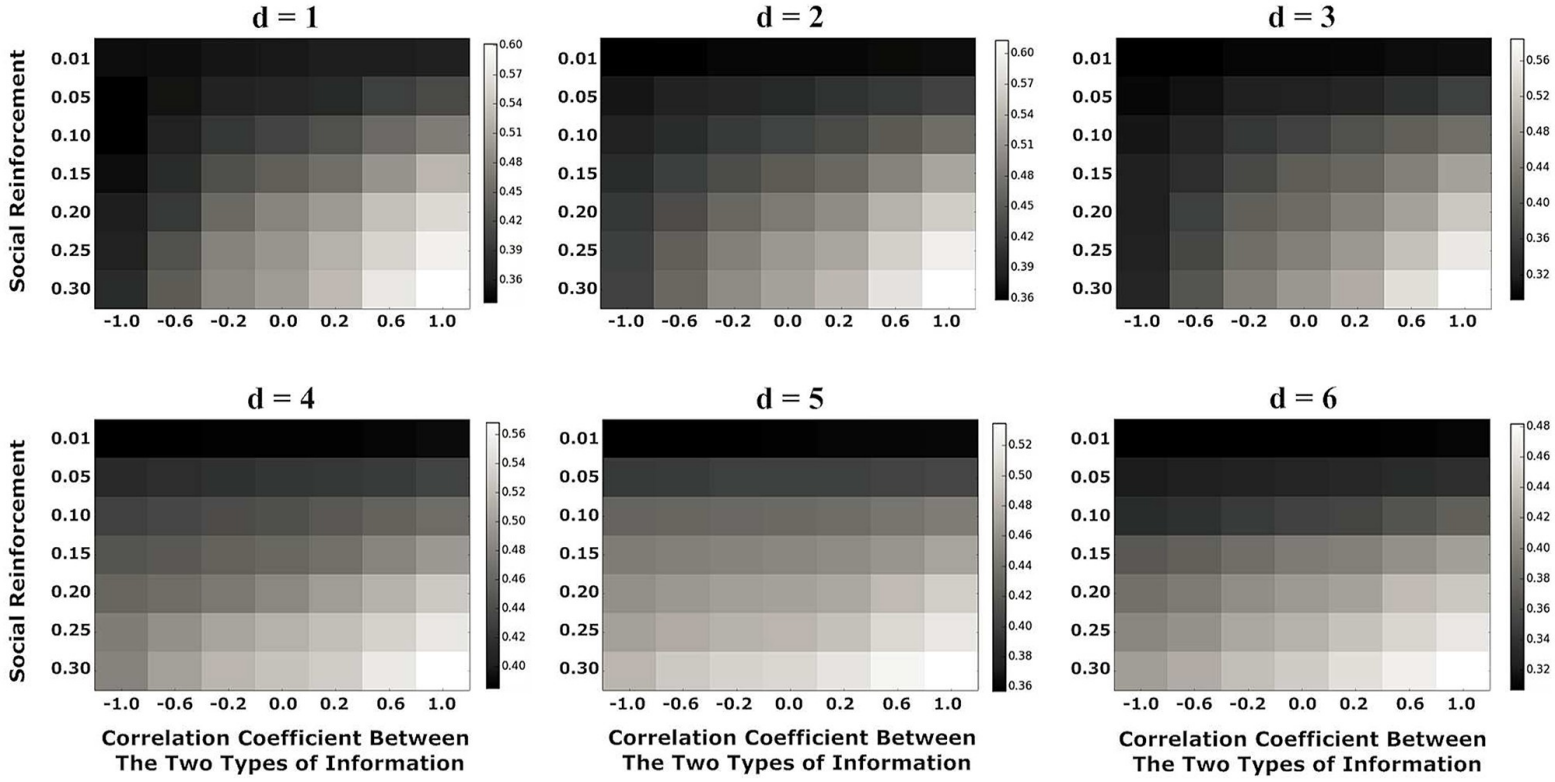
The influence of the distance (*d*) between the two information sources on information spreading when correlation coefficient (*β*) and social reinforcement (*c*) take different values.

We first analyzed the impact of the distance (*d*) between the two information sources on information spreading when correlation coefficient (*β*) and social reinforcement (*c*) take different values. Fig 4 presents the following results.

1. When *d* is fixed, the increase of *β* or *c* will enhance the coverage of the information spreading.
2. When *β* and *c* are fixed, with the increase of *d*, the coverage of information spreading will be reduced (*β* > 0) or enhanced (*β* < 0), respectively.
3. With the increases of *d*, *c* has a more significant influence on the information spreading than *β*.

**Fig 5:**
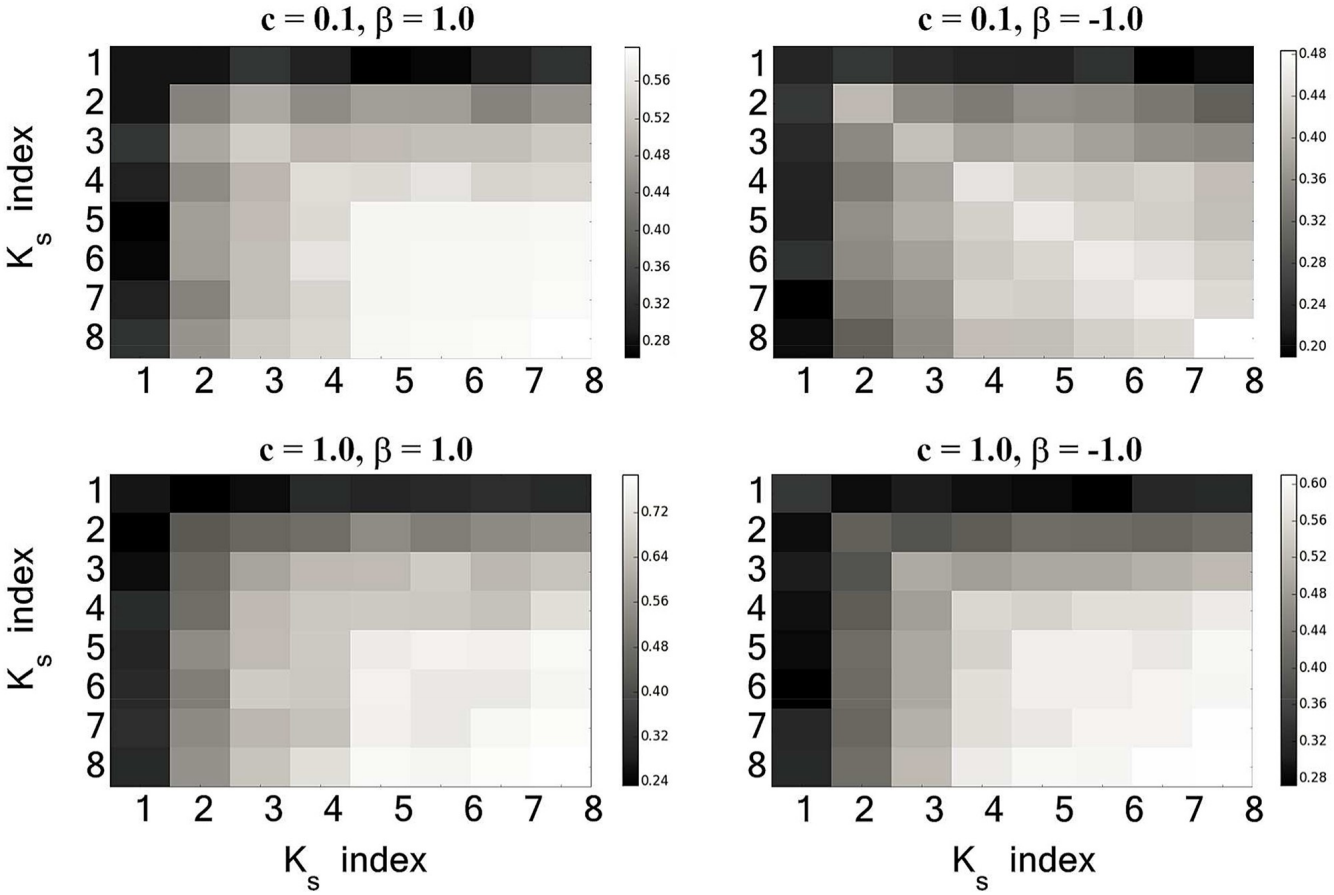
The influence of two information sources at different *K*-*shell* layers on information spreading when *β* and *c* are fixed. The horizontal and vertical coordinates represent the *K*_*S*_ values of the *K*-*shell* layer where the two information sources are located, respectively.

Next, we separately analyzed the influence of *K*-*shell* layers and community structure on information spreading. Firstly, we considered the effect of two information sources at different *K*-*shell* layers on information spreading. In Fig 5, it can be found that the larger the *K*_*S*_ value of the *K*-*shell* layers where two information sources are located, the wider the coverage of information spreading is. However, we found that there may be other factors besides *K*-*shell* layers that affect information spreading.

**Fig 6:**
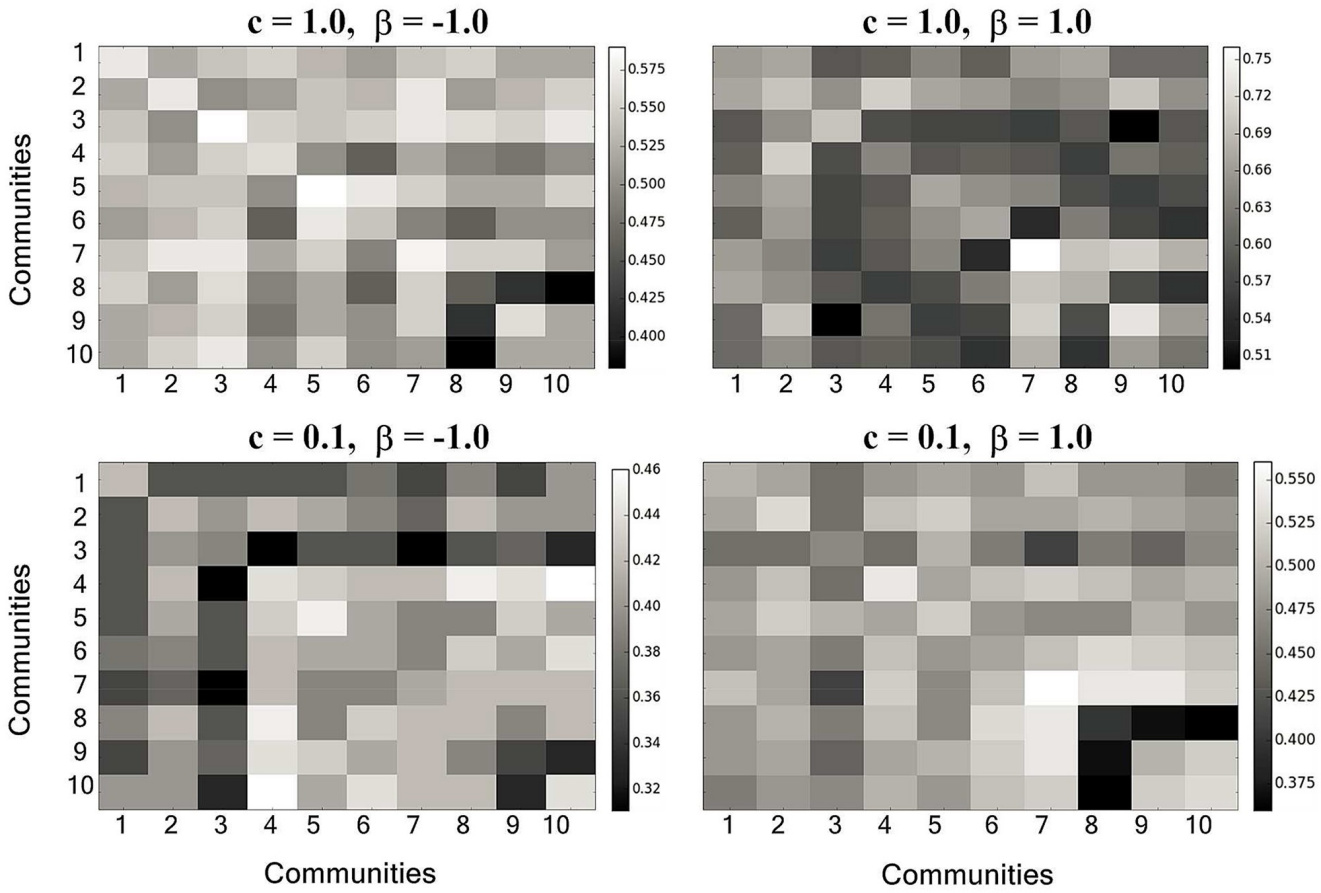
The influence of two information sources at different communities on information spreading when *β* and *c* are fixed. The horizontal and vertical coordinates represent the communities where the two information sources are located, respectively.

Then, we analyzed the influence of two information sources on information spreading when they were in different communities. In Fig 6, no specific relationships between information spreading and communities can be observed when only considering the influence of community structure.

Finally, we analyzed and compared the influence of various factors on information spreading. First, we compared the influence of the distance between two information sources and *K*-*shell* layers on information spreading.

As shown in Fig 7,

1. No matter how far the distance between the two information sources is, the larger the *K*_*S*_ value is, the wider the coverage of information spreading will be.
2. In the same *K*-*shell* layer, when the value of *c* is small, no specific relationships between information spreading and the distance between the two information sources can be observed; however, when the values of *c* and *β* are large, the smaller the distance between the two sources is, the wider the coverage of information spreading will be.

**Fig 7:**
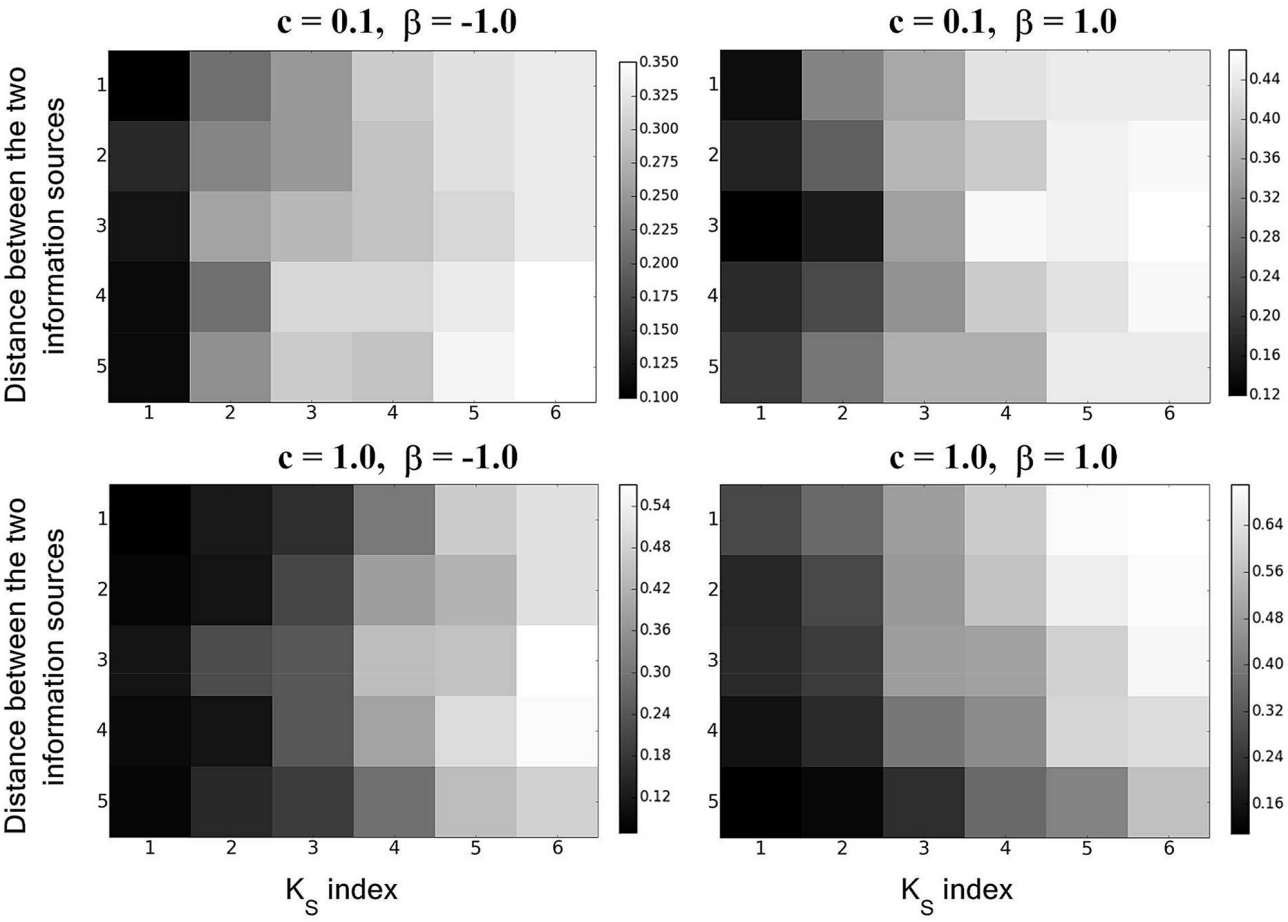
The influence of the distance (*d*) between the two information sources and *K*-*shell* layers on information spreading when *β* and *c* are fixed. The horizontal coordinates represent the *K*_*S*_ value of the *K*-*shell* layer where two information sources are located in the same *K*-*shell* layer, and the vertical coordinates represent distance (*d*) between two information sources.

Finally, we compared the influence of community structure and *K*-*shell* layers on information spreading.

**Fig 8:**
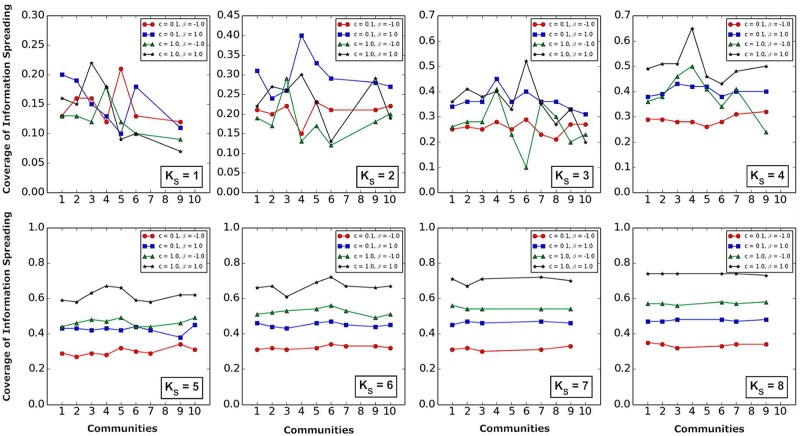
The influence of community structure and *K*-*shell* layers on information spreading when *K*_*S*_ value, *β* and *c* are fixed. The horizontal coordinates represent the community where two information sources are located in the same community. The vertical coordinates represent the coverage of information spreading.

**Fig 9:**
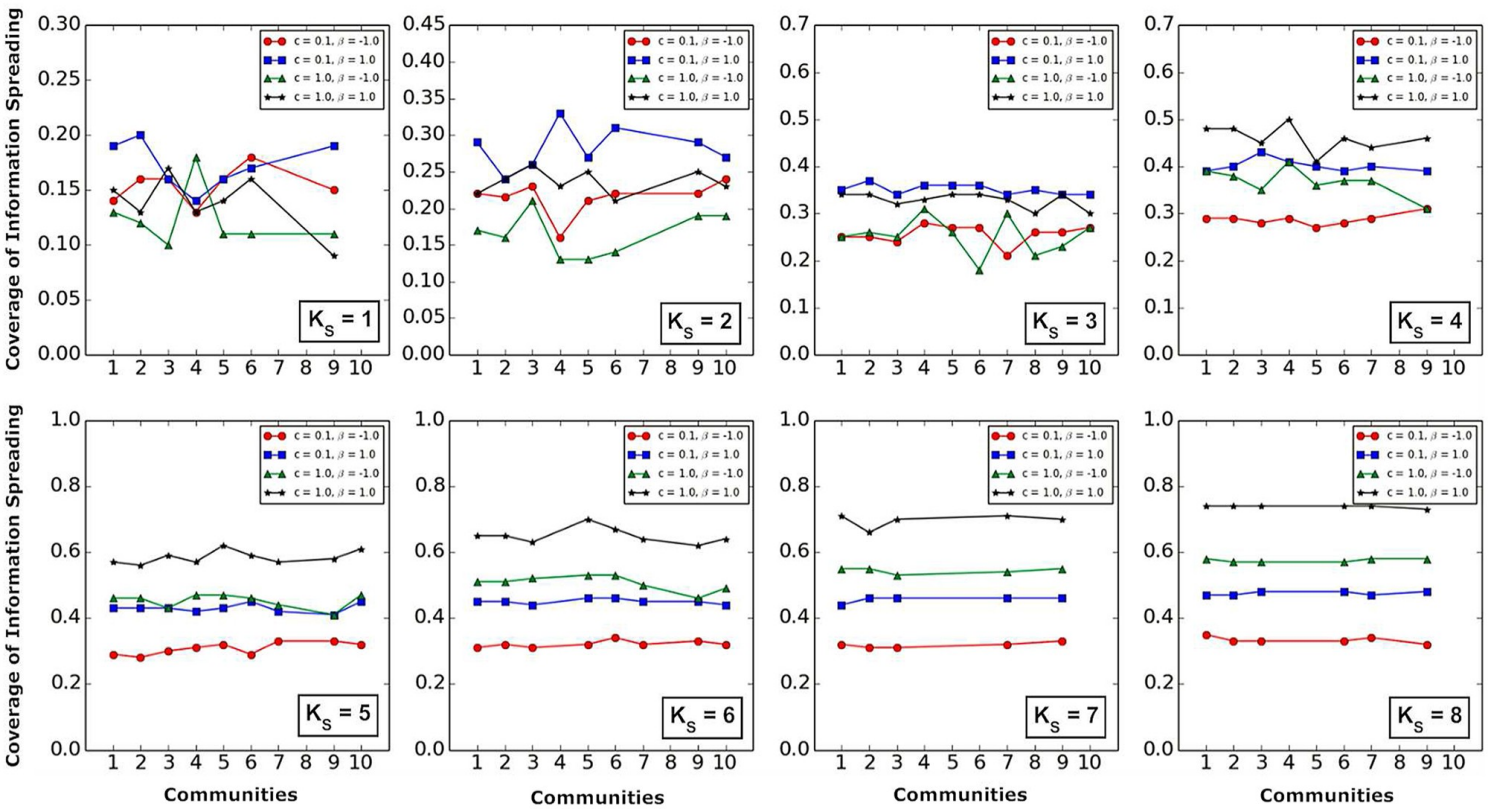
The influence of community structure and *K*-*shell* layers on information spreading when *K*_*S*_ value, *β* and *c* are fixed. The horizontal coordinates represent the communities where two information sources are located in different communities, and *i* (*i* ∈ 1, 2, 3, 4, 5, 6, 7, 8, 9, 10) represents that one information source is in community *i*, and the other information source is in one of the remaining communities. The vertical coordinates represent the coverage of information spreading.

Figs 8 and 9 show that,

1. Whether two information sources are located in the same community (Fig 8) or in different communities (Fig 9), the larger the *K*_*S*_ values of the *K*-*shell* layers where two information sources are located, the smaller the influence of community structure on information spreading is.
2. When the *K*_*S*_ value is higher (*K*_*S*_ = 5, 6, 7, 8) (Figs 8 and 9), *c* has a more significant impact on information spreading than *β*.

**Fig 10:**
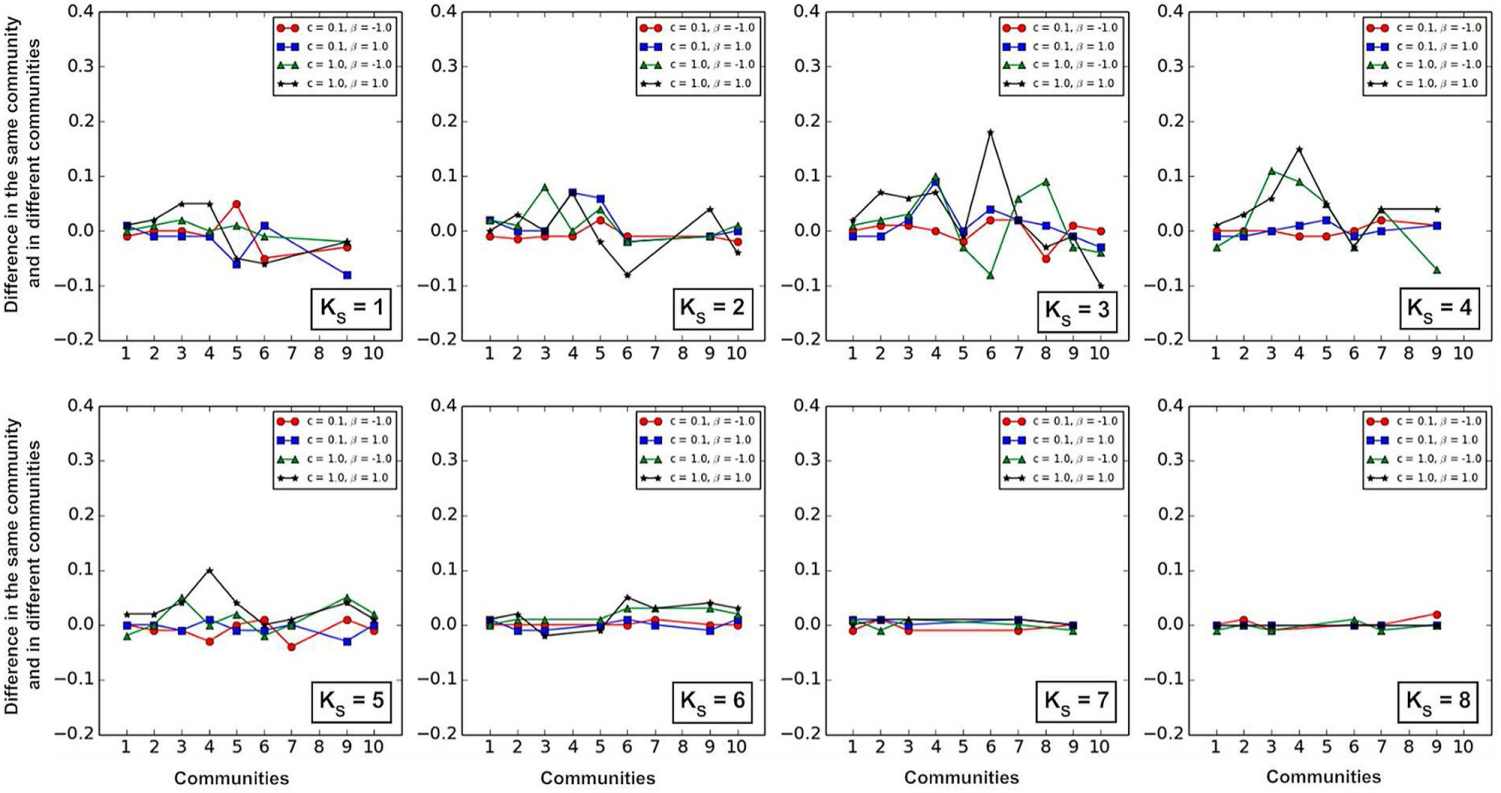
Comparison of the spread of two information sources in the same community and in different communities when *K*_*S*_ value, *β* and *c* are fixed. For example, we compare the two cases: (i) two information sources within the same community 1; (ii) one of two information sources in the community 1, and the other in other community (2, 3, 4, 5, 6, 7, 8, 9, or 10).

We further compared the spread of two information sources in the same community and in different communities. Fig 10 shows that when the *K*_*S*_ value is larger (*K*_*S*_ = 6, 7, 8), the coverage of information spreading shows very little differences.

**Fig 11:**
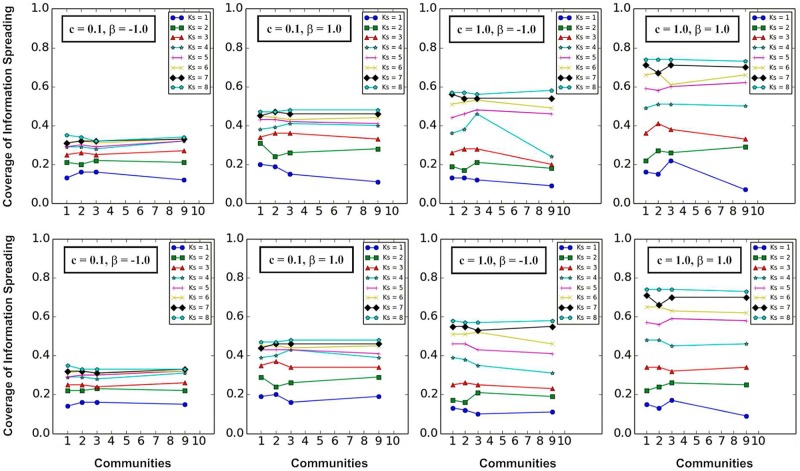
The influence of community structure and *K*-*shell* layers on information spreading when *β* and *c* are fixed. The horizontal coordinates represent the community where two information sources are located in the same community (the top 4 subfigures). The horizontal coordinates represent the communities where two information sources are located in different communities (the bottom 4 subfigures), and *i* (*i* ∈ 1, 2, 3, 4, 5, 6, 7, 8, 9, 10) represents that one information source in a community *i*, and the other information source is in one of the remaining communities. The vertical coordinates represent the coverage of information spreading.

In Fig 11, it can be found that whether the two information sources are in the same community or different communities, the higher *K*_*S*_ values corresponds to the wider coverage of information spreading.

In order to test and verify the universality of the conclusions obtained above, we conduct simulation experiments on more networks based on the spread model in this paper (see supplementary information). At the same time, we also found that the above conclusions were valid in the other three networks.

## Discussion

In order to better understand the spreading mechanisms and the key affecting factors of information spreading in social networks, this paper proposed a model of information spreading that considered five spreading mechanisms: memory effects, social reinforcement, non-redundancy, human heterogeneity, and attentional decline. By various simulation experiments, we analyzed the influence of social reinforcement, correlation coefficient between the two types of information, distance between the two information sources in the network, community structure and *K*-*shell* layers on information spreading. The main conclusions were as follows.

1. *K*-*shell* layers have the greatest impact on information spreading.
  The greater the *K*_*S*_ values are, the wider the coverage of information spreading will be, which is consistent with the results of a previous study [18]: The most efficient spreaders are those located within the core of the network, as identified by the *K*-*shell* decomposition analysis.
2. Distance between the two information sources, correlation coefficient between two types of information, social reinforcement and community structure affect the information spreading.
  - When the distance between two information sources is fixed, the increase in the values of *β* or *c* will enhance the coverage of the information spreading.
  - When the values of *β* and *c* are fixed, with the increase of distance between two information sources, the coverage of information spreading will be reduced (*β* > 0) or enhanced (*β* < 0), respectively.
  - With the increases of distance between the two sources of information, *c* has a more significant influence on the information spreading than *β*.
  - When the values of *β*(*β* > 0) and *c* are fixed, with the increase of distance between the two sources, the coverage of information spreading will be reduced. This result is consistent with the conclusion of Kitsak et al. [18]: When considering the distance between the two information sources, the distance is the crucial factor that determines the extent of the spreading. However, it is surprising that in the same *K*-*shell* layer, when the value of *c* is smaller, there is no obvious correlation between distance between the two information sources and information spreading.
  - When the *K*_*S*_ value is higher, community structure does not affect the spread of information. This result is just contrary to the conclusion of Nematzadeh et al. [17], who reported that community structure has certain effects on information spreading.

In order to be closer to the real information spreading process, this paper has established the information spreading model as authentically as possible from two aspects: (1) the background network is a real social network, or a network generated by a network evolution model closer to the real social network structure; (2) the information spreading model closer to the real situation is established. Then we comprehensively and systematically analyzed various factors affecting information spreading, including factors in spreading model (such as *β* and *c*) and factors in network structure (such as *K*_*S*_ values and communities). Therefore, the analysis results of these factors can help to predict the trend of information spreading in the network: when two information sources are located in the *K*-*shell* layer with larger *K*_*S*_ value, the coverage of information spreading will be larger (*β* > 0), or smaller (*β* < 0). In addition, through the results of this study, we can further find effective strategies to control information spreading.

## Conclusion

The results in this paper will facilitate a better understanding of the patterns of information spreading in social networks and clarify the key factors that affect multiple information spreading, which can provide some guidance for achieving the maximum information spreading (such as fads, innovations, collective actions and opinions) as well as stopping the transmission of viruses and controlling the spread of rumors. In addition, the information spreading model proposed in this paper can be applied to the research on the spread of more types of information in the network.

## Supporting information

S1 Appendix Data sources of three background networks (*G*_1_, *G*_2_, *G*_3_ and *G*_4_). (PDF)

S1 File The simulation results of information spreading on network *G*_2_. (PDF)

S2 File The simulation results of information spreading on network *G*_3_. (PDF)

S3 File The simulation results of information spreading on network *G*_4_. (PDF)
