# Supplementary material for: Factors affecting the spread of multiple information in social networks
Source: PLoS One. 2019 Dec 12;14(12):e0225751. doi: 10.1371/journal.pone.0225751 (PMC6907769; doi:10.1371/journal.pone.0225751)
Supplement: S1 Appendix — (PDF) [file pone.0225751.s001.pdf]

S1 Appendix. Data sources of three background networks (G_1, G_2 , G_3 and G_4)

(1) **G_1**: Data set from Alex Arenas personal web page:
http://deim.urv.cat/~alexandre.arenas/data/welcome.htm

(2) **G_2**: Generated by social network evolution model [1], and two adjustable parameters p_1 and p_2 satisfies: p_1 = 0.1, p_2 = 0.2; and the number of nodes in G_2 is 500.

(3) **G_3**: Generated by social network evolution model [1], and two adjustable parameters p_1 and p_2 satisfies: p_1 = 0.2, p_2 = 0.3; and the number of nodes in G_3 is 500.

(4) **G_4**: A real social network (Facebook friendships), which contain friendship data of Facebook users, comprises 63,731 nodes and 817,035 edges. The data set of network can be downloaded from the following websites:
http://konect.uni-koblenz.de/networks/facebook-wosn-links

[1] Zhiqiang Zhu, A novel method of generating tunable network topologies for social simulation, Journal of Statistical Mechanics: Theory and Experiment, 2018, 073410.
