# Supplementary material for: Factors affecting the spread of multiple information in social networks
Source: PLoS One. 2019 Dec 12;14(12):e0225751. doi: 10.1371/journal.pone.0225751 (PMC6907769; doi:10.1371/journal.pone.0225751)
Supplement: S1 File — (PDF) [file pone.0225751.s002.pdf]

S1 File. The simulation results of information spreading on network G_2

Fig S1_1: analysis of the influence of the distance (***d***) between the two information sources on information spreading;

Fig S1_2: analysis of the influence of ***K-shell* layers** on information spreading;

Fig S1_3: analysis of the influence of **community structure** on information spreading;

Fig S1_4: analysis of the influence of the distance (***d***) between the two information sources and ***K-shell* layers** on information spreading;

(**Fig S1_5**, **Fig S1_6**, **Fig S1_7**): analysis of the influence of **community structure** and ***K-shell* layers** on information spreading.

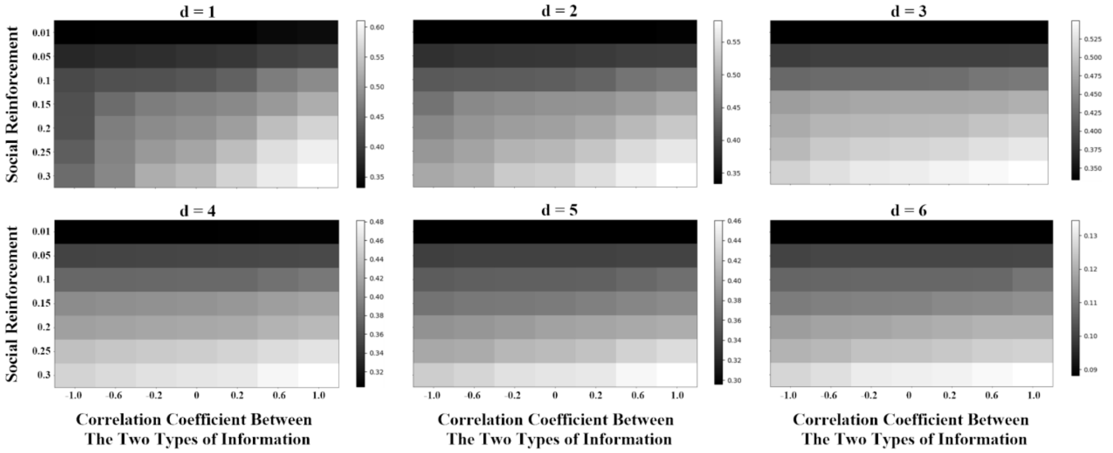

Fig S1_1. The influence of the distance (*d*) between the two information sources on information spreading when correlation coefficient (*β*) and social reinforcement (*c*) take different values.

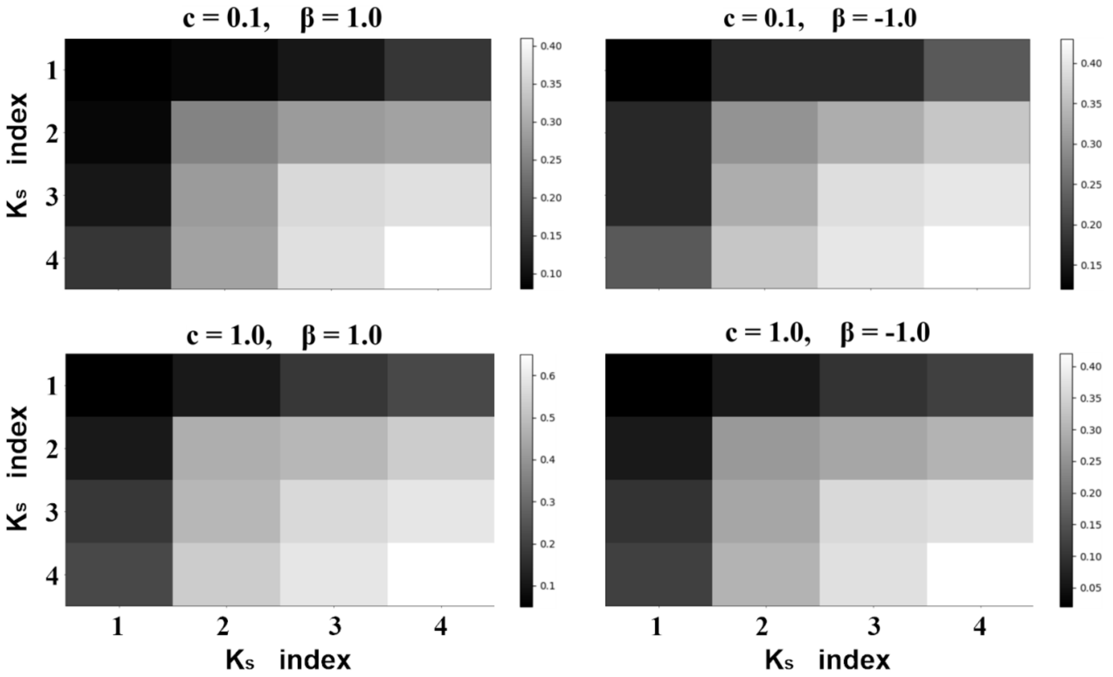

Fig S1_2. The influence of two information sources at different *K-shell* layers on information spreading when *β* and *c* are fixed. The horizontal and vertical coordinates represent the *Ks* values of the *K-shell* layer where two information sources are located, respectively.

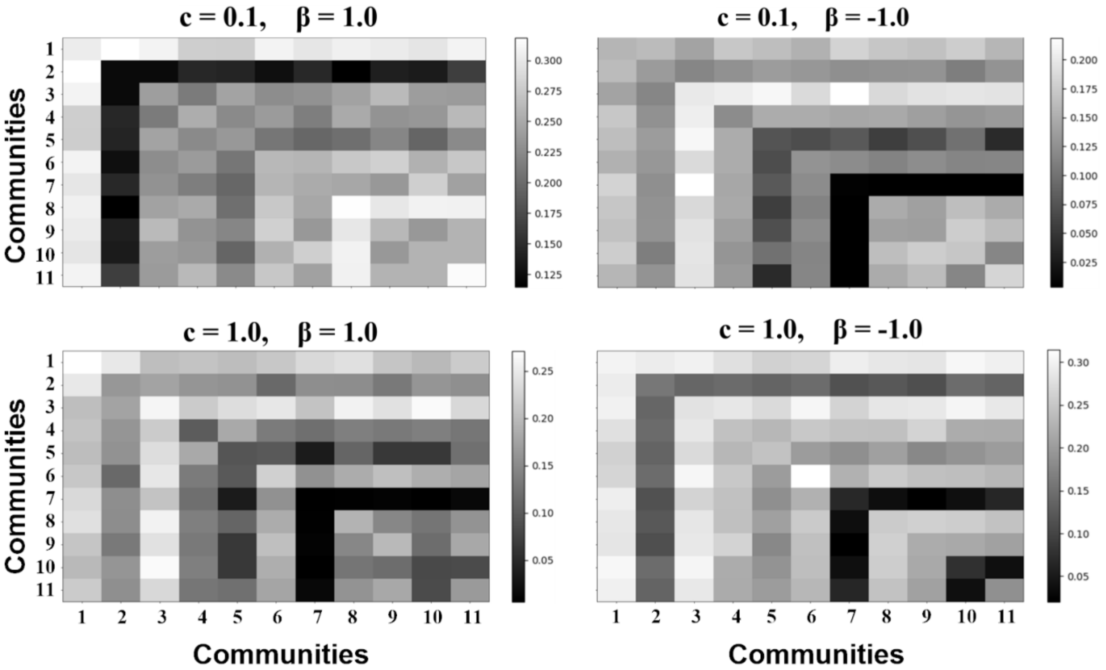

Fig S1_3. The influence of two information sources at different communities on information spreading when *β* and *c* are fixed. The horizontal and vertical coordinates represent the communities where two information sources are located, respectively.

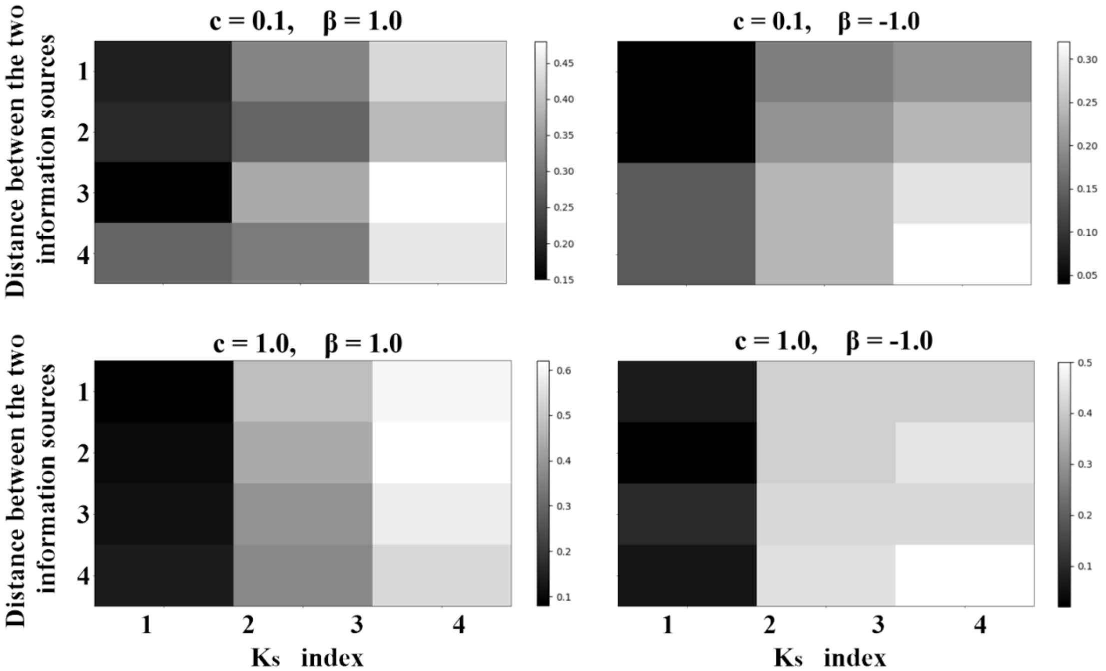

Fig S1_4. The influence of the distance (*d*) between the two information sources and *K-shell* layers on information spreading when *β* and *c* are fixed. The horizontal coordinates represent the *Ks* value of the *K-shell* layer where two information sources are located in the same *K-shell* layer, and the vertical coordinates represent distance (*d*) between two information sources.

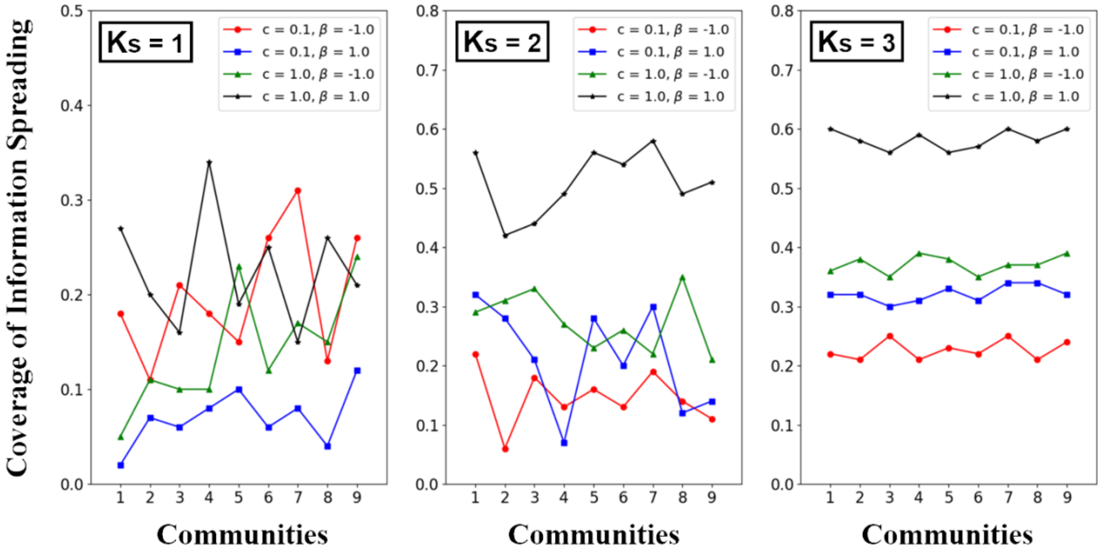

Fig S1_5. The influence of community structure and *K-shell* layers on information spreading when *Ks* value, *β* and *c* are fixed. The horizontal coordinates represent the community where two information sources are located in the same community. The vertical coordinates represent the coverage of information spreading.

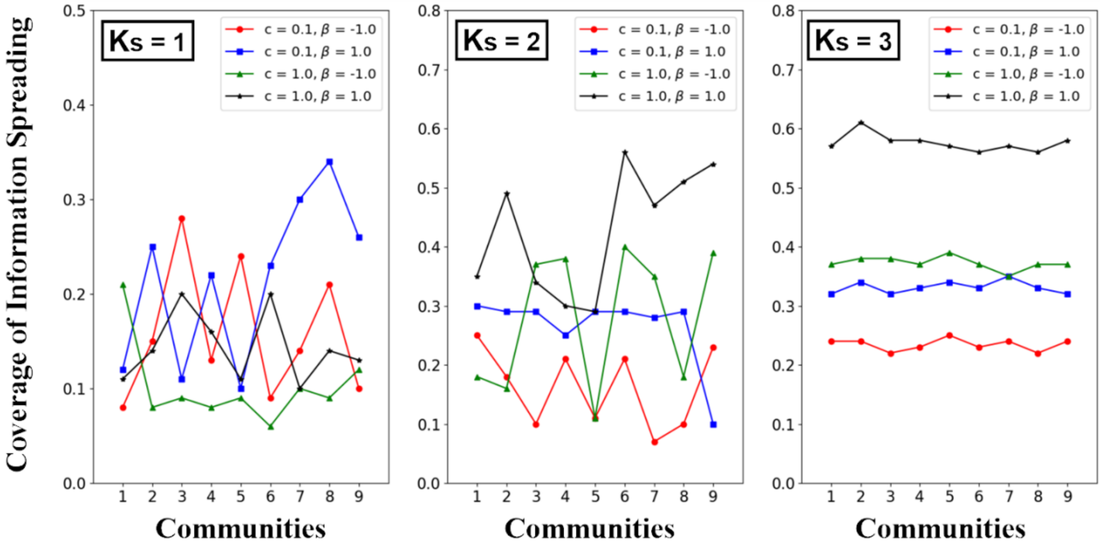

Fig S1_6. The influence of community structure and *K-shell* layers on information spreading when *Ks* value, *β* and *c* are fixed. The horizontal coordinates represent the communities where two information sources are located in different communities, and *i* (*i* ∈{1,2,3,4,5,6,7,8,9}) represents one information source is in community *i*, and the other information source is in one of the remaining communities. The vertical coordinates represent the coverage of information spreading.

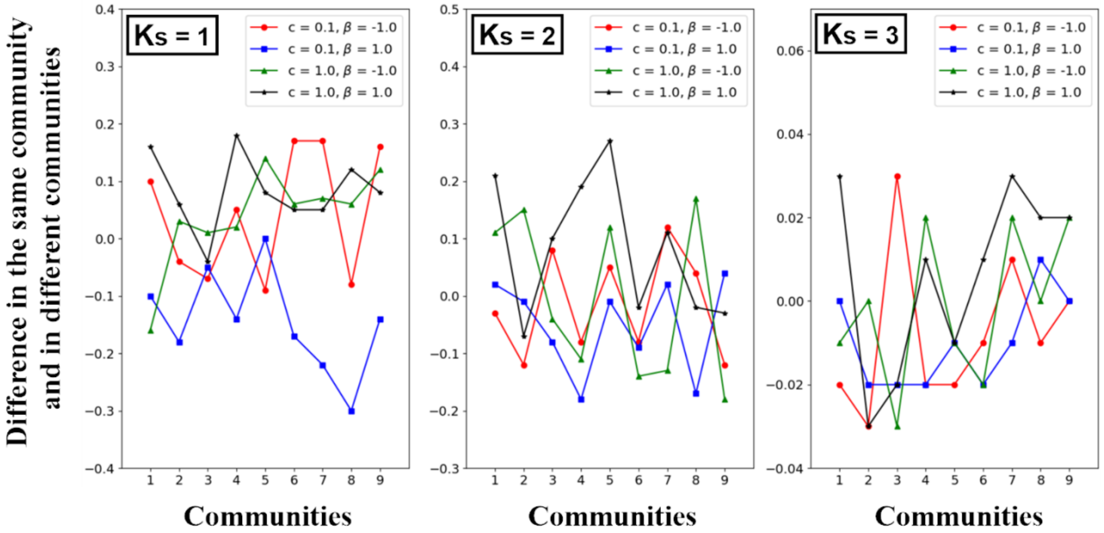

Fig S1_7. Comparison of the spread of two information sources in the same community and in different communities when *Ks* value, *β* and *c* are fixed. For example, we compare the two cases: (i) two information sources within the same community 1; (ii) one of two information sources in the community 1, and the other information source in other community (2, 3, 4, 5, 6, 7, 8, or 9).
